# Mechanical compression induces VEGFA overexpression in breast cancer via DNMT3A-dependent miR-9 downregulation

**DOI:** 10.1038/cddis.2017.73

**Published:** 2017-03-02

**Authors:** Baek Gil Kim, Ming-Qing Gao, Suki Kang, Yoon Pyo Choi, Joo Hyun Lee, Ji Eun Kim, Hyun Ho Han, Seong Gyeong Mun, Nam Hoon Cho

**Affiliations:** 1 Department of Pathology, Yonsei University College of Medicine, Seoul, South Korea; 2 Brain Korea 21 Plus Project for Medical Science, Yonsei University College of Medicine, Seoul, South Korea; 3 Severance Biomedical Science Institute (SBSI), Yonsei University College of Medicine, Seoul, South Korea; 4 Global 5-5-10 System Biology, Yonsei University, Seoul, South Korea; 5 College of Veterinary Medicine, Northwest A&F University, Yangling, Shaanxi, China

## Abstract

Tumor growth generates mechanical compression, which may trigger mechanotransduction in cancer and stromal cells and promote tumor progression. However, very little is known about how compression stimulates signal transduction and contributes to tumor progression. In the present study, we demonstrated that compression enhances a tumor progression phenotype using an *in vitro* compression model, and validated the results from the *in vitro* model with high- and low-compressed breast cancer tissues. Mechanical compression induced miR-9 downregulation by DNMT3A-dependent promoter methylation in the MDA-MB-231 and BT-474 breast cancer cell lines and in cancer-associated fibroblasts. The overexpression of miR-9 target genes (*LAMC2*, *ITGA6,* and *EIF4E*) was induced by miR-9 downregulation, which eventually enhanced vascular endothelial growth factors production. Demethylation and decompression could reverse compression-induced miR-9 downregulation and following overexpression of miR-9 target genes and *VEGFA.*

The increase of cellularity in a restricted tissue space causes mechanical compression. Therefore, tumor growth generates mechanical compression, which may affect the signal transduction of cancer and stromal cells and promote tumor progression. This mechanotransduction may be a component of the cancer signaling axis. However, little is known about how mechanical compression contributes to tumor progression.

In a living organism, various mechanical stresses naturally occur and can modulate cellular signaling.^1, 2, 3, 4, 5, 6, 7, 8, 9^ Unlimited proliferation is a fundamental property of cancer.^10^ Therefore, tumor proliferation in a restricted tissue space increases mechanical compression, which may happen in almost all solid tumors. It was reported that increased mechanical compression acts in the interior and periphery of tumors.^11^ The solid stress by tumor proliferation is known to be a cause for the increase of compressive force in tumor tissue.^12^ In addition to tumor proliferation, an excessive deposition of extracellular matrix (ECM) contributes to solid stress by stiffening soft tissues.^13^ The degree of tissue stiffness is associated with breast cancer malignancy.^14, 15^ Because uncontrolled proliferation and tissue stiffness are common features of cancer, it follows that compression-induced mechanotransduction may be important for tumor progression. It was demonstrated that mechanical compression can induce cancer cell migration by stimulating the formation of leader cells.^16^ Nonetheless, the signaling pathways downstream of mechanical stress are largely unknown in cancer.

Previously, we found that laminin-332 and integrin-*α*6*β*4 are overexpressed in the margin between the tumor and fibrotic stroma in breast invasive ductal carcinoma (IDC).^17^ The margin is the periphery where compression is induced by tumor growth; thus, laminin-332 and integrin-*α*6*β*4 overexpression may be induced by compression and may play an important role in breast cancer progression. Laminin-332 promotes tumorigenesis, tumor invasion, and survival.^18, 19^ Integrin-*α*6*β*4 is also involved in cancer progression.^20^ Most importantly, the binding of laminin-332 to integrin-*α*6*β*4 contributes to angiogenic signaling. It was shown that integrin alpha6beta4 enhances EIF4E activity, leading to the increased production of vascular endothelial growth factors (VEGF) in breast cancer cells.^21^ Interestingly, *LAMC2* (the gene encoding the gamma subunit of laminin-332), *ITGA6* (the gene encoding integrin alpha6), *ITGB4* (the gene encoding integrin beta4), and *EIF4E* have microRNA-9 (miR-9) binding sites in their 3'-UTRs (microRNA.org and TargetScan), and miR-9 is downregulated in advanced breast cancer.^22, 23^ The connection between miR-9 and compression has not been reported. However, a mechanical stress is capable of altering miR expression.^24^ miR-18a is induced by a mechanical stress and leads to a reduction in phosphatase and tensin homolog (PTEN) expression.^25^ Taken together, it is plausible that compression can induce miR-9 downregulation, which would lead to the upregulation of miR-9 target genes

In this study, we demonstrated that mechanical compression downregulates miR-9 expression in cancer-associated fibroblasts (CAFs), which are major component contributing to matrix stiffness, and breast cancer cells by using an agarose-scaffolded alginate bead culture for a static three-dimensional (3D) compression model. In addition, we also showed that compression-induced miR-9 downregulation triggers and enhances a signaling axis for VEGFA.

## Results

### Mechanical compression downregulated miR-9 expression in breast cancer

**Figure 1:**
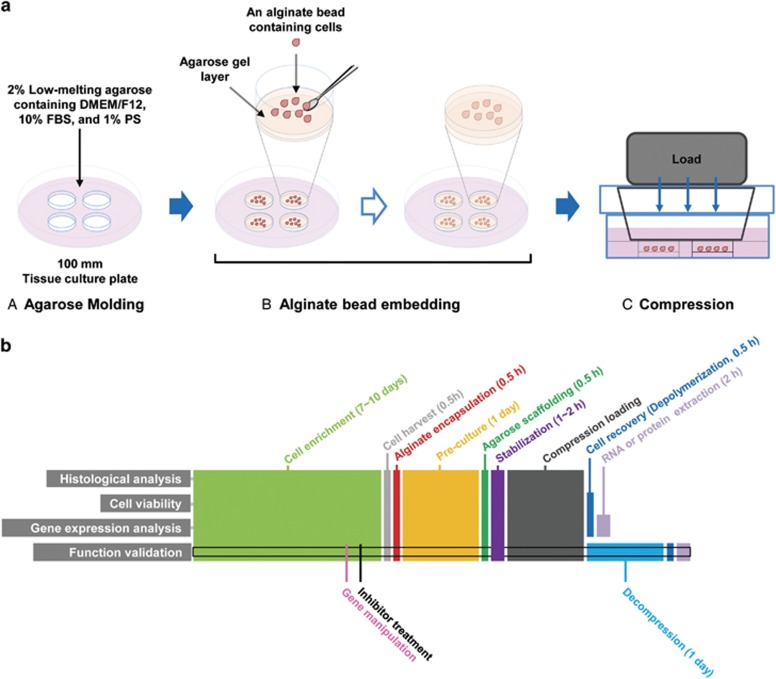
Illustration of the agarose-scaffolded alginate bead culture for the 3D static compression model and timeline for experiments. (**a**) Procedure for the agarose-scaffolded bead culture for compression. (A) Agarose molding. Twenty-five milliliter of the low-melting agarose containing DMEM/F12, 10% FBS, and 1% PS was poured into a 100 mm tissue culture plate. To embed the CAF-alginate beads, the agarose gel was punched using a sterile 15 ml tube. (B) Alginate bead embedding. A thin layer of agarose was made on the bottom of a punched hole. The cell-alginate beads pre-cultured for ECM deposition a day before embedding were laid on the agarose layer. The rest part around the cells-alginate beads was filled with agarose gel. (C) Loading static compression. Compression was loaded using the cube filled with iron ball bearings. An empty cube was loaded for control. (**b**) Timeline for the experiments. The samples for experiments were generally obtained in the same procedure except for histological analysis and function validation. For histological analysis, the alginate beads containing cells were fixed with 4% paraformaldehyde, embedded in paraffin, sectioned into 5-*μ*m-thick sections, and stained with hematoxylin and eosin. For function validation, inhibitor treatment was started 1 days before cell harvest. For the sample preparation of decompressed cells, the cells were incubated for 1 day without compression

miR-9 downregulation in advanced breast cancer may be associated with the mechanical compression (CF) generated by cancer cell proliferation. To confirm whether compression induces miR-9 downregulation in breast cancer, CAFs and breast cancer cell lines [MCF7, BT-474, MDA-MB-231, and SK-BR-3: representative cell lines for luminal A, luminal B, Claudin-low (triple negative), and Her2 amplification of human breast cancer, respectively^26^] were compressed with 0.5, 1, 2, 5, and 10 relative compression units (RCUs, 1 RCU=~0.773 kPa, the compression value of a native tumor microenvironment^16^) using an agarose-scaffolded alginate bead culture model. Agarose-scaffolded alginate bead culture was firstly developed for this study, since the previous compression models using agarose gel requires heating or chaotropic salts for agarose depolymerization.^27, 28^ Heat and chaotropic salts impair the integrity of RNA and protein.^29, 30^ Alginate is one of the widely used biomaterials for cell culture^31^ and is easy to polymerize and depolymerize. However, alginate beads were not strong enough to endure the compression of native tumor microenvironment. To make alginate beads endurable to compression, they were embedded within an agarose gel (Figure 1a). The suitability of our model was evaluated as Supplementary Figure S1.

**Figure 2:**
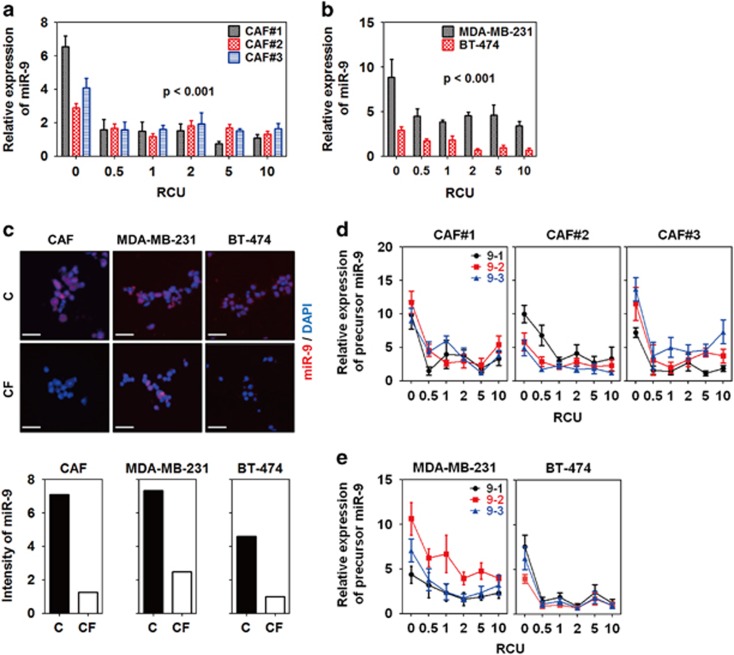
Mechanical compression downregulated miR-9 expression in breast cancer. Compression-induced miR-9 expression of (**a**) CAFs and (**b**) breast cancer cells (MDA-MB-231 and BT-474). CAFs were isolated from three IDC patients. For compression, the weights of each indicated RCU were loaded on the cells for 24 h. Statistical significance was determined using a control (**c**) *versus* compressed sample *t*-test. All compressed samples had a statistical significance of *P*<0.001. (**c**) Fluorescent images of compression-induced miR-9 downregulation in CAFs and breast cancer cells. For detecting miR-9, the cells were compressed with one RCU for 24 h in the presence of a Cy5-conjugated miR-9-5p probe, then isolated from the alginate beads by depolymerization, fixed with 4% paraformaldehyde, smeared onto a gelatin-coated microscope slide, and then mounted with a coverslip and mounting medium. Scale bar, 200 *μ*m. Fluorescence images were quantified by measuring red-colored area using Image J. Compression-induced precursor miR-9 expression of (**d**) CAFs and (**e**) MDA-MB-231 and BT-474. All data are represented as mean±S.D.

In our model, miR-9 expression was significantly decreased by compression in CAFs, MDA-MB-231, and BT-474 at all RCUs (Figures 2a and b). On the other hand, miR-9 expression was not changed or upregulated by compression in MCF7 and SK-BR-3 (Supplementary Figure S2A). To confirm compression-induced miR-9 downregulation, CAFs, MDA-MB-231, and BT-474 were compressed with 1 RCU for 24 h in the presence of a Cy5-conjugated miR-9 probe. As shown in Figure 2c, the fluorescence of miR-9 was diminished by compression in CAFs, MDA-MB-231, and BT-474 compared with the controls. miR-9 is derived from three precursors, mir-9-1, mir-9-2, and mir-9-3. Therefore, the expression of miR-9 precursors was further evaluated. Similar to miR-9 expression, mir-9-1, -2, and -3 were downregulated by compression in CAFs, MDA-MB-231, and BT-474 at all RCUs (Figures 2d and e).

### Compression-induced miR-9 downregulation was associated with DNMT3A-dependent methylation

**Figure 3:**
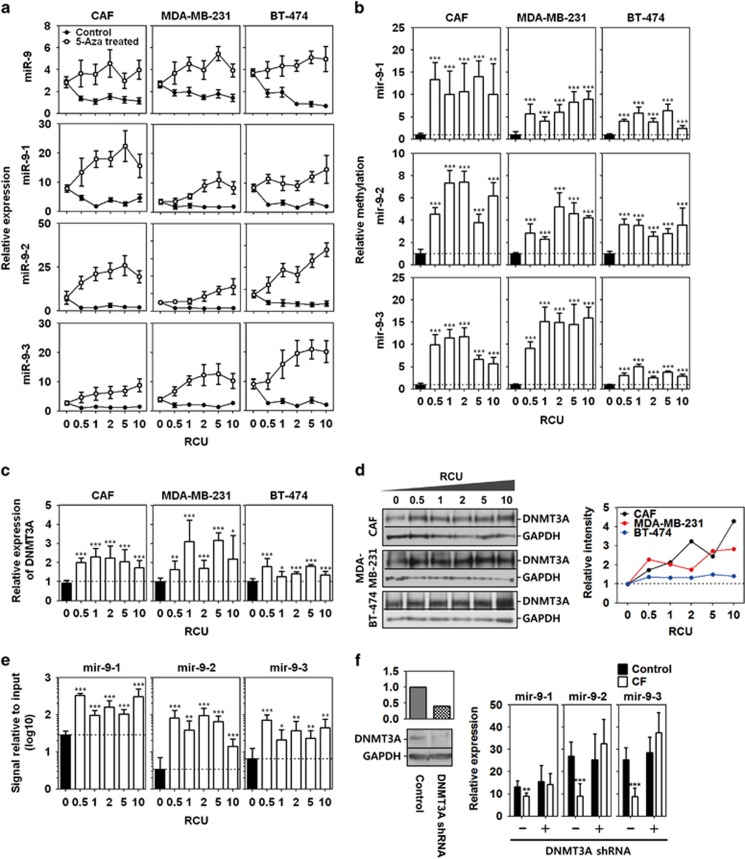
Compression-induced miR-9 downregulation via DNMT3A-dependent methylation. (**a**) Compression-induced miR-9 and its precursor expression of CAF, MDA-MB-231, and BT-474 in the presence or absence of 5-azacytidine (5-aza). Cell-alginate beads were pretreated with 10 *μ*M 5-aza for 1h before compression. (**b**) Methylation-specific PCR analysis of putative promoters of pre-mir-9-1, pre-mir-9-2, and pre-mir-9-3. Methylated DNA was calculated by normalizing to unmethylated DNA using the ΔΔCt method. Compression-induced expression of (**c**) *DNMT3A* mRNA and (**d**) DNMT3A protein in CAF, MDA-MB-231, and BT-474. CAF1 was used as a representative for CAF. The intensity of western bands were quantified using Image J. Each band was normalized with GAPDH, and then presented as the relative intensity value of control *versus* compressed sample. (**e**) Chromatin immunoprecipitation (Chip) assay to examine DNMT3A binding to the promoter regions of miR-9 precursors. The CAF1 compressed at the RCU of 1 for 24 h were used for Chip assay, and then DNMT3A-bound DNA was analyzed using real-time PCR. Relative binding to DNMT3A was calculated using the ΔΔCt method. The Ct values of DNMT3A-bound DNA were normalized to those of input DNA. (**f**) DNMT3A-dependent downregulation of miR-9 precursors. The wilde-type and DNMT3A shRNA overexpressing MDA-MB-231 were exposed to 1 RCU for 24 h to induce miR-9 downregulation. Data are represented as mea±S.D. Statistical significance was determined using a control- *versus* compressed sample *t*-test. *, **, and *** represent *P-*values of 0.01 to 0.05, 0.001–0.01, and<0.001, respectively

miR-9 downregulation in breast cancer is known to be associated with hypermethylation.^22^ Therefore, a compression-induced regression of miR-9 and its precursors was investigated in the absence or presence of the methylation inhibitor 5-azacytidine (5-aza). As shown in Figure 3a, the expression of miR-9 and its precursors in 5-aza-treated CAFs, MDA-MB-231, and BT-474 was higher than in untreated cells at the indicated RCUs. 5-aza treatment inhibited compression-induced miR-9 downregulation (i.e., increased miR-9 expression) at all RCUs.

Promoter hypermethylation can lead to gene repression. Thus, compression may be associated with the promoter hypermethylation of miR-9 precursors. To test this hypothesis, the region 2 kb upstream of each miR-9 precursor locus was analyzed to determine the putative promoter regions. Four, one, and two CpG islands were identified in the 2 kb region upstream of mir-9-1, mir-9-2, and mir-9-3, respectively (Supplementary Figure S3). For methylation-specific qPCR analysis, methylated and unmethylated DNA-specific primer sets were designed at the third CpG island of mir-9-1, the first CpG island of mir-9-2, and the second island of mir-9-3 since a largest number of CpG sites are located in the CpG islands. The relative methylation of the precursor miR-9 promoters was calculated by normalizing to unmethylated DNA using the ΔΔCt method. As shown in Figure 3b, the methylation of the mir-9-1 putative promoter was raised over 10-fold in CAFs, and 3–10-fold in in MDA-MB-231 and BT-474 following compression. A similar, but less degree of methylation was observed in the mir-9-2 putative promoter region (3–8-fold increase). The methylation of the mir-9-3 putative promoter was also increased by compression in CAFs, MDA-MB-231, and BT-474. In BT-474, putative promoter regions of precursor miR-9 were methylated following compression, but to a lesser extent than in CAFs and MDA-MB-231.

*De novo* promoter methylation is catalyzed by the DNMT3 family of enzymes. Therefore, it was examined whether compression induces the overexpression of DNMT3A, 3B, and 3 l. *DNMT3A* mRNA was upregulated by compression at all the RCUs in CAF, MDA-MB-231, and BT-474 (Figure 3c). DNMT3A protein was also overexpressed following compression (Figure 3d). However, unlike *DNMT3A*, *DNMT3B* mRNA was not elevated by compression, but rather downregulated at some RCUs (Supplementary Figure S4A). *DNMT3 L* mRNA was not generally changed by compression at RCUs in CAF and MDA-MB-231, whereas was significantly decreased in BT-474 (Supplementary Figure S4B). The DNMT3B and 3L proteins showed very low expression and little difference between RCUs (Supplementary Figure S4C). Unlike CAF, MDA-MB-231 and BT-474, as shown in Supplementary Figure S5A, MCF7 and SK-BR-3 showed a significant downregulation of compression-induced *DNMT3A* mRNA expression. Similar to mRNA, the expression of DNMT3A protein was generally decreased in MCF7 by compression except for 1 RCU, whereas it was decreased in SK-BR-3 at the RCUs of 0.5, 5, and 10 (Supplementary Figure S5D).

To confirm whether the hypermethylation of the putative promoter regions was induced by DNMT3A, chromatin immunoprecipitation with anti-DNMT3A antibody was performed. As shown in Figure 3e, DNMT3A bindings to the putative promoter regions of miR-9 precursors were higher in compressed cells than uncompressed control cells. In the MDA-MB-231 stably overexpressing DNMT3A shRNA, compression did not induce the downregulation of precursors of miR-9 (Figure 3f).

### Compression-induced miR-9 downregulation was reversed by decompression

**Figure 4:**
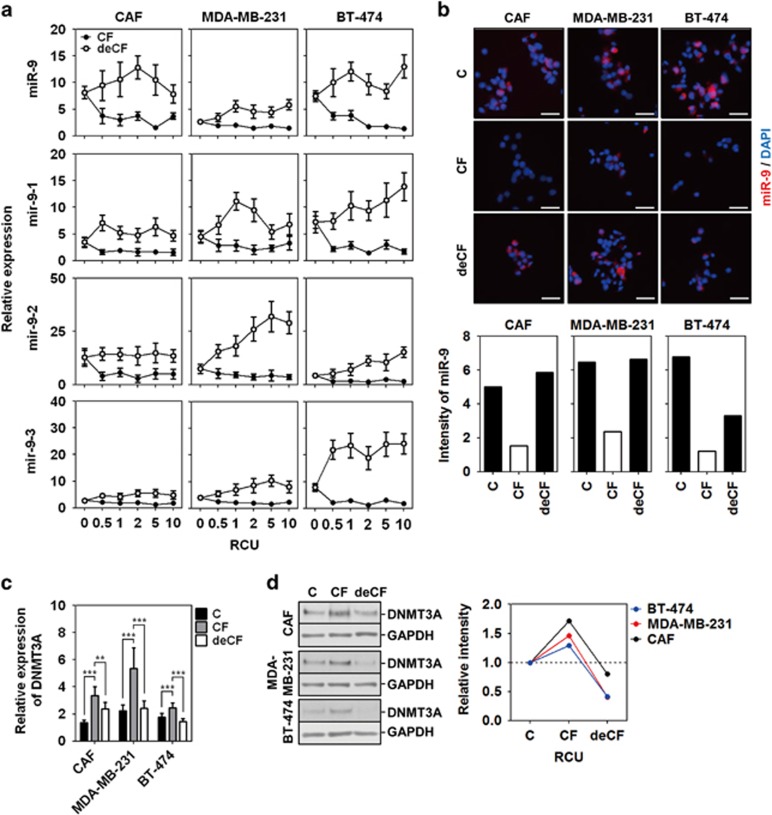
Compression-induced miR-9 downregulation was reversed by decompression. (**a**) The effect of decompression (deCF) on miR-9 and its precursor expression. (**b**) Fluorescent images of miR-9 expression by decompression. Scale bar, 200 *μ*m. (**c**) *DNMT3A* mRNA expression and (**d**) DNMT3A protein expression by decompression. CAF1, MDA-MB-231, and BT-474 were compressed with 1 RCU for 24 h. For decompression, the cells were cultured for an additional 24 h without compression. Data are represented as mean±S.D. ** and *** represent *P*-values of 0.001–0.01, and<0.001, respectively

To further validate the influence of compression on miR-9 expression, cells were compressed for 24 h and then incubated for an additional 24 h without compression (i.e., decompression, or deCF). As shown in Figure 4a, miR-9 expression was upregulated by decompression in the CAF, MDA-MB-231, and BT-474, compared with the controls which were compressed for 24 h but without decompression. Similar to miR-9, the expression of each precursor miR-9 was also increased by the decompression in CAF, MDA-MB-231, and BT-474 compared with the controls. The red fluorescent miR-9 probe intensity, which had been weakened by compression, recovered by decompression (Figure 4b). Compression-induced miR-9 downregulation was induced by DNMT3A-mediated hypermethylation (Figures 3b and e). Therefore, restored miR-9 expression by decompression may be coupled with DNMT3A downregulation. As shown in Figures 4c and d, an upregulated expression of DNMT3A mRNA and protein by compression was downregulated by decompression.

### Compression-induced miR-9 downregulation was associated with VEGF expression

**Figure 5:**
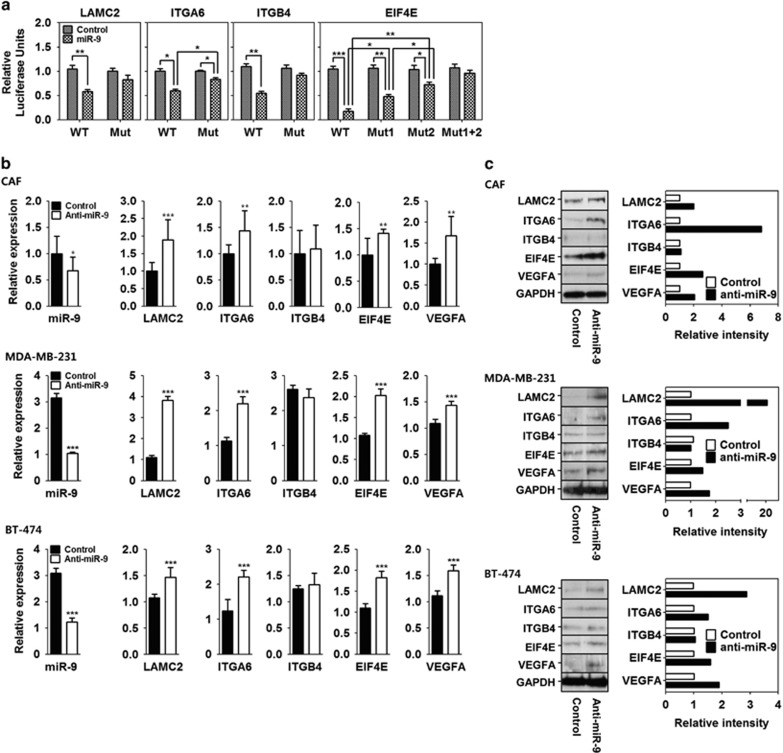
Compression-induced miR-9 downregulation was associated with VEGFA expression. (**a**) Luciferase reporter gene assay using the *LAMC2*, *ITGA6*, *ITGB4*, and *EIF4E* 3'-UTRs. (**b**) mRNA expression and (**c**) protein expression of *LAMC2*, *ITGA6*, *ITGB4*, *EIF4E*, and *VEGFA* in the CAF1, MDA-MB-231, and BT-474 transfected with anti-miR-9. For the negative control, a control miR was transfected. Data are represented as mean±S.D. Statistical significance was determined using a control- *versus* miR-9- or anti-miR-9-transfected-sample *t*-test. *, **, and *** represent *P*-values of 0.01–0.05, 0.001–0.01, and<0.001, respectively

Compression-induced miR-9 downregulation may contribute to angiogenesis, since miR-9 downregulation is observed in advanced breast cancer and tumor progression is dependent of angiogenesis. It was previously reported that the signaling axis consisting of laminin-332, integrin-*α*6*β*4, and EIF4E enhances the expression of VEGFs.^21^ Laminin-332 and integrin-*α*6*β*4 are also known to be overexpressed in the margin between tumor and fibrotic stoma, where compression is induced by tumor growth in breast IDC.^17^ *LAMC2* encodes the gamma subunit of laminin-332, *ITGA6* and *ITGB4* encode the subunits of integrin-*α*6*β*4, and *EIF4E* encodes the EIF4E protein. Interestingly, *LAMC2*, *ITGA6*, *ITGB4*, and *EIF4E* have miR-9-binding sequences in their 3'-UTRs (microRNA.org and TargetScan). Accordingly, we hypothesized that compression-induced miR-9 downregulation would result in the upregulation of *LAMC2*, *ITGA6*, *ITGB4*, and *EIF4E*. To determine whether miR-9 binds to the 3'-UTRs of *LAMC2*, *ITGA6*, *ITGB4*, and *EIF4E*, wild and seed sequence deletion mutants of each 3'-UTRs were cloned into the pGL3 control vector (Supplementary Figure S6), and then miR-9 binding to the 3'-UTRs was measured by luciferase activity assay. The luciferase activities of wild-type 3'-UTRs of *LAMC2*, *ITGA6*, *ITGB4*, and *EIF4E* were all significantly decreased by miR-9 co-transfection compared with those of the mutant 3'-UTRs (Figure 5a).

Next, we determined whether miR-9 downregulation leads to the upregulation of *LAMC2*, *ITGA6*, *ITGB4*, *EIF4E, VEGFA* in CAF, MDA-MB-231, and BT-474. In the CAF, MDA-MB-231, and BT-474 transfected with anti-miR-9, the mRNA and protein expression of *LAMC2, ITGA6, EIF4E,* and *VEGFA* were significantly upregulated, whereas those of *ITGB4* was not (Figures 5b and c).

### Compression-induced expression of miR-9 target genes was suppressed by methylation inhibition and decompression

**Figure 6:**
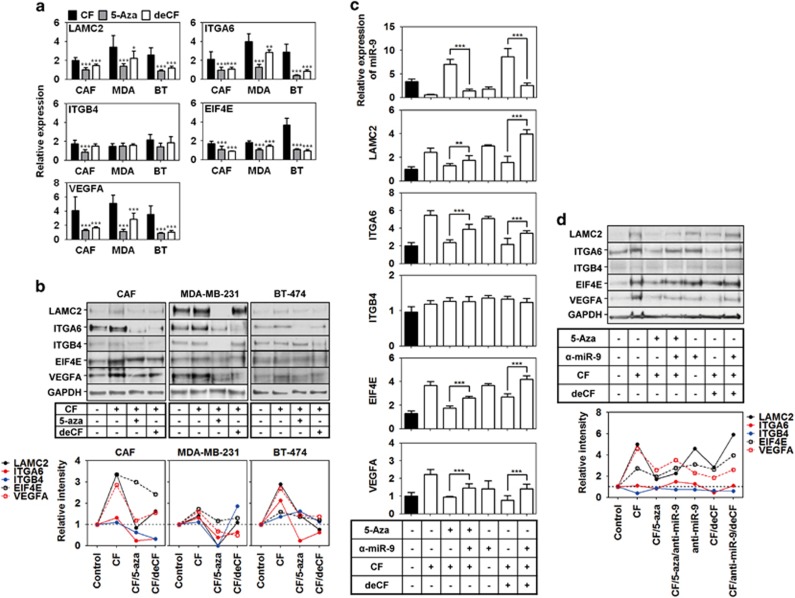
Compression-induced expression of miR-9 target genes and VEGFA was suppressed by methylation inhibition and decompression. The effect of methylation inhibition and decompression on (**a**) mRNA expression and (**b**) protein expression of miR-9 target genes and VEGFA in CAF, MDA-MB-231, and BT-474. (**c**) miR-9 dependent downregulation of LAMC2, ITGA6, EIF4E, and VEGFA by methylation inhibition and decompression in CAF. CAF1 was used as a representative for CAF. The cells were compressed with the RCU of 1 for 24 h in the absence or presence of 10 *μ*M of 5-azacytidine. For decompression, the cells were cultured for additional 24 h without compression. Data are represented as mean±S.D. ** and *** represent *P*-values of 0.001–0.01 and <0.001, respectively

miR-9 downregulation induced the upregulation of *LAMC2*, *ITGA6*, *EIF4E*, and *VEGFA* expression (Figures 5b and c). Since compression-induced miR-9 downregulation was reversed by methylation inhibition (5-aza treatment) and decompression, the expression of *LAMC2*, *ITGA6*, *EIF4E*, and *VEGFA* may be also regulated by methylation inhibition and decompression. To confirm this assumption, we compressed CAF, MDA-MB-231, and BT-474 at the RCU of 1 for 24 h in the absence or presence of 5-aza, or further incubated for an additional 24 h without compression, and then analyzed mRNA expression. As shown in Figure 6a, a compression-induced upregulation of *LAMC2, ITGA6, EIF4E, and VEGFA* was decreased by 5-aza treatment and decompression in CAF, MDA-MB-231, and BT-474. On the other hand, *ITGB4* expression was generally not decreased by 5-aza treatment and decompression. It was decreased by 5-aza treatment only in CAF. Like mRNA expression, LAMC2, ITGA6, EIF4E, and VEGFA protein expression was increased by compression, but decreased by 5-aza treatment or decompression in CAFs, MDA-MB-231, and BT-474 (Figure 6b). ITGB4 protein expression was largely unaffected by compression, decompression, or 5-aza treatment.

To confirm whether decreased expression of LAMC2, ITGA6, EIF4E, and VEGFA by 5-aza treatment or decompression was dependent on miR-9 expression, CAF was tranfected with anti-miR-9, and then compressed in the presence or absence of 5-aza at the RCU of 1. For decompression, the compressed CAF was further incubated for 24 h without compression. As shown in Figures 6c and d, a decreased expression of LAMC2, ITGA6, EIF4E, and VEGFA by 5-aza treatment or decompression was reversed by anti-miR-9.

### miR-9 and its target gene expression are associated with the compressive state of the tissue

**Figure 7:**
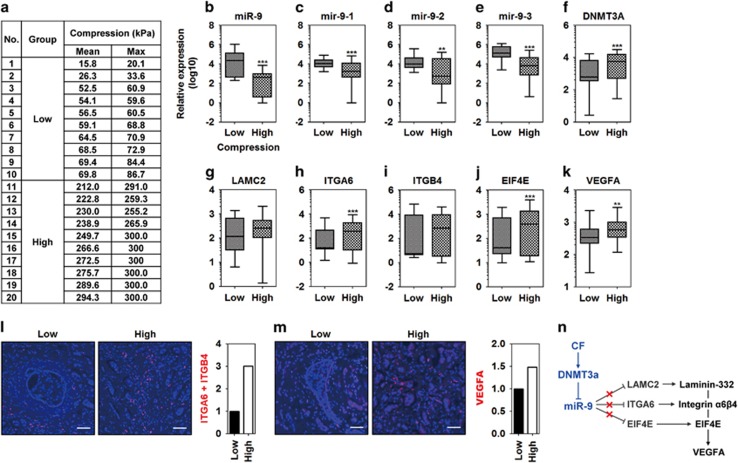
miR-9 and its target gene expression are associated with the compressive state of the tissue. (**a**) Table of compression values of low- and high-compressed breast cancer tissues. Tissues were obtained from breast cancer patients after measuring compression by shear-wave elastography. (**b**) miR-9 expression in the low- and high-compressed tissues of breast cancer. Expression of (**c**) mir-9-1, (**d**) mir-9-2, and (**e**) mir-9-3 in the low- and high-compressed tissues of breast cancer. mRNA expression of (**f**) *DNMT3A*, (**g**) *LAMC2*, (**h**) *ITGA6*, (**i**) *ITGB4*, (**j**) *EIF4E*, and (**k**) *VEGFA* in the low- and high-compressed tissues of breast cancer. **, and *** represent *P*-values of 0.001–0.01 and <0.001, respectively. Data are represented as the mean±S.D. Statistical significance was determined using the nonparametric test of a low- *versus* high-compressed tissue. PLA of (**l**) integrin-*α*6*β*4, and (**m**) VEGFA. The PLA assay for ITGA6 was performed using the double recognition of rabbit anti-ITGA6 and mouse anti-ITGB4 antibodies. The PLA assay for VEGFA was performed using the single recognition of mouse anti-VEGFA antibody. Fluorescence images were quantified by measuring red-colored area using Image J. (**n**) A signal transduction pathway for VEGFA by compression-induced miR-9 downregulation

To further validate the results of the 3D static compression model, we evaluated gene expression of low- and high-compressed breast cancer tissues from patients. Tissue stiffness or elasticity is significantly correlated with tumor cellularity,^32^ and increased cellularity by tumor expansion generally leads to high compressive state in tissue via solid stress.^12^ Therefore, low- and high-compressed tissues were classified using the mean compression values obtained by Shear-wave elastography (SWE, low-compression group, <100 kPa; high-compression group, >250 kPa; Figure 7a). miR-9 and its precursors were downregulated in high-compressed tissues compared with low-compressed tissues (Figures 7b and e). In contrast, *DNMT3A* expression was upregulated in high-compressed tissues compared with low-compressed tissues (Figure 7f). Among miR-9 target genes, *LAMC2* expression was not associated with the compressed state of the tissues (Figure 7g). *ITGA6* expression was increased in the high-compressed tissues compared with low-compressed tissues (Figure 7h). *ITGB4* expression was not associated with the compressive state of tissues (Figure 7i). *EIF4E* and *VEGFA* expression was increased the in high-compressed tissues compared with low-compressed tissues (Figures 7j and k, respectively). The increased expression of *ITGA6* and *VEGFA* in high-compressed tissues was similar to the increased expression observed in CAFs, MDA-MB-231, and BT-474. Therefore, the expression of ITGA6 and VEGFA was investigated by *in situ* proximity ligation assay (PLA) on tissue sections. As shown in Figure 7l, a more amount of red-fluorescence was observed in high-compressed tissue than low-compressed one when performing double recognition for ITGA6 with ITGB4. Like ITGA6, a more amount of red-fluorescence was observed in high-compressed tissue than low-compressed one upon performing single recognition for VEGFA (Figure 7m).

## Discussion

In our experimental model, mechanical compression induced miR-9 downregulation in CAFs, MDA-MB-231, and BT-474 (Figures 2a and b), whereas it did not induce miR-9 downregulation in MCF7 and SK-BR-3 (Supplementary Figure S2A). There may be two possible assumptions for this heterogeneous response of breast cancer cells to compression. One is cell-type specificity. For instance, stress-activated p53 induces a heterogeneous response in a cell-type-specific manner.^33^ Another is the different expression status of growth factors such as estrogen receptor (ER), progesterone receptor (PR), and Her2. All the receptors anchored in the cell membrane can function as mechanoreceptors.^34^ A ligand-independent function of ER has an essential role in osteocyte and osteoblast mechanotransduction.^35^ In CAFs, MDA-MB-231, and BT-474, compression-induced miR-9 downregulation contributed to VEGFA overexpression by inducing overexpression of LAMC2, ITGA6, and EIF4E. Interestingly however, compression-induced miR-9 upregulation in MCF7 and SK-BR-3 may contribute to VEGF production through another mechanism. Ma *et al* showed that miR-9 downregulated E-cadherin, which resulted in *β*-catenin signaling promoting VEGF production in MCF7.^36^

Compression-induced downregulation of miR-9 was reversed after decompression for 24 h (Figure 4). In our study, miR-9 downregulation resulted from promoter hypermethylation by compression-induced DNMT3A overexpression. DNMT3A is required for *de novo* methylation, but it is not needed for the maintenance of methylation.^37, 38^ Hence, miR-9 reversion cannot be explained by decompression-induced DNMT3A downregulation. DNMT3A downregulation may be indirectly associated with miR-9 promoter demethylation. The balance in the dynamics of DNA methylation and demethylation is an epigenetic mechanism for gene expression.^39, 40^ Therefore, decompression-induced DNMT3A downregulation can result in the imbalance between methylation and demethylation, which leads to demethylation of miR-9 promoter. With DNMT3A downregulation, decompression may activate demethylases such as the Ten-eleven translocation methylcytosine dioxygenase (TET) or the methylation-sensitive transcription factor to lead miR-9 upregulation such as Yin Yang 1 (YY1).^41^ Kangaspeska *et al.*^42^ reported transient cyclical methylation and demethylation of CpG dinucleotides in the estrogen (E2)-responsive *pS2* gene. This may be an evidence of reversible methylation state.

The compression model with cell-agarose constructs has been developed in order to study the cells exposed to periodic compression and decompression such as osteoblasts and odontoblasts. The model should be suitable to investigate compression-induced mechanotransduction, but it has an obvious disadvantage in sampling. For extracting RNA and protein, agarose has to be depolymerized by heating or adding chaotropic salts, which adversely affect sample quality.^27, 28^ To figure out the problem, we encapsulated cells with alginate before embedding them into agarose. Alginate is one of the good biomaterials for 3D cell culture and easily depolymerized by EDTA.^27^ Unfortunately, since alginate bead was not strong enough to endure the compression of native tumor microenvironment, we scaffolded the cell-alginate beads with agarose. By using agarose-scaffolded cell-alginate bead constructs, we could recover cells easily after loading compression and then extract RNA and protein using common methods. Tse *et al.*^16^ reported that the compression more than 5.8 mm Hg (1 RCU) triggered apoptosis and only 40% of cells viable at 58 mm Hg (10 RCU). However, in our model, there was little difference in apoptosis between RCUs (Supplementary Figure S1D). This discrepancy may be caused by difference between 2D and 3D cell culture environment. We encapsulated cells with alginate for 3D cell culture, whereas Tse *et al.* cultured cells on the membrane of transwell chamber. 3D environment has two merits compared with 2D. 3D is more similar to *in vivo* situation than 2D, and 3D is more advantageous in ECM deposition. ECM is known as a transmitter of mechanical stress.^34, 43^ As shown in Supplementary Figure S1E, ECM deposition around cells was observed in alginate beads.

Here, we showed that compression induces miR-9 downregulation via DNMT3A-dependent promoter methylation, which leads to the upregulation of miR-9 target genes (*LAMC2*, *ITGA6*, and *EIF4E*) thus potentiating signal transduction for VEGFA expression in CAFs and breast cancer cells. We also showed that decompression can restore miR-9 expression and subsequent downregulation of miR-9 target genes. Therefore, compression-induced miR-9 downregulation may be an important mechanism for tumor angiogenesis.

## Materials and Methods

### Tissue acquisition

For CAF isolation, human breast tumor tissues were obtained from three IDC patients. Tissue stiffness is along with tumor growth-induced solid stress,^44^ and the solid stress increases compression force.^12^ Therefore, to study compression-dependent gene expression, low- and high-compressed breast tumor tissues were obtained from 20 breast cancer patient tissues after measuring compression by shear-wave elastography (SWE) using the Aixplorer US system (SuperSonic Imagine, Aix-en-Provence, France): 10 for low-compression below the mean value of 100 kPa and 10 for high-compression over the mean value of 200 kPa. All patients donating the tissues underwent surgery at Severance Hospital of the Yonsei University Health System, Seoul, South Korea. The protocol for the research was approved by the Severance Hospital Ethics Committee (IRB number 4-2008-0383). All participants signed consent forms and were informed of tissue use for comprehensive experiments of breast cancer.

### Isolation of cancer-associated fibroblasts and cell cultures

CAFs were isolated as previously described.^45^ Briefly, tissue from early-stage IDC (stage 1) that was less than 10 mm in diameter was sliced and then digested overnight with a collagenase preparation (ISU ABXIS; Seoul, South Korea). Digested tissue was filtered through a 70 *μ*m cell strainer (SPL Life Science; Pocheon-si, South Korea). Cells were separated by Ficoll gradients, washed with PBS, resuspended with DMEM/F12 cell culture medium containing 20% (v/v) fetal bovine serum (FBS), 100 IU/ml penicillin, and 100 *μ*g/ml streptomycin (Gibco BRL; Grand Island, NY, USA) and cultured at 37 °C in a humidified incubator containing 5% CO_2_. The fibrotic nature of the isolated cells was confirmed by microscopic determination of morphology and immunofluorescence characterization using the antibodies against vimentin (Abcam; Cambridge, UK), cytokeratin (Dako; Glostrup, Denmark) and cytokeratin 5 (Novocastra; Newcastle upon Tyne, UK). Breast cancer cell lines (BT-474, MCF7, SK-BR-3, MDA-MB-231) were purchased from Korean Cell Line Bank (Seoul, South Korea) (authenticated using morphology and STR profiling) and cultured with DMEM cell culture medium containing 10% (v/v) fetal bovine serum (FBS), 100 IU/ml penicillin, and 100 *μ*g/ml streptomycin at 37 °C in a humidified incubator containing 5% CO_2_.

### Compression assay and sample preparation

Compression assays were performed as previously described.^46^ Briefly, for making the alginate beads containing cells, pellets of CAFs, MDA-MB-231, BT-474, MCF7, or SK-BR-3 were resuspended in 0.5% alginate solution to yield 5 × 10^6^ cells/ml. Using a syringe with a 21-gauge needle, the alginate solution was dropped into 102 mM CaCl_2_ for polymerization.^47^ The cell-alginate beads were washed with PBS twice, and then cultured at 37 °C in a humidified CO_2_ incubator for 24 h. In this step, cells were stabilized and deposited ECM around them. For compression, pre-cultured cell-alginate beads were embedded with 2% low-melting agarose into an agarose mold as shown in Supplementary Figure 1A, stabilized in medium for 1 h, and then compressed with RCUs for 24 h. One RCU was 5.8 mmHg (~0.773 kPa),^16^ the compression value of a native tumor microenvironment. An empty cube was placed on control samples to make the same diffusion condition as other compressed ones. For decompression, the cell-alginate beads were compressed for 24 h and then incubated for an additional 24 h without compression. For sample preparation, the cell-alginate beads were depolymerized with 55 mM EDTA, washed with PBS twice, centrifuged at 250 r.c.f. for 3 min, and were then used immediately in experiments or were stored at −80 °C. Our compression model was validated as follows. The deformation of gel is considered as an indicator for the transfer of a mechanical stress to cells in compression models.^48^ As shown in Supplementary Figures S1A and S1B, the deformation of agarose gel and alginate bead was proportional to the degree of compression. To confirm whether compression was transferred to cells, the expression of c-Jun was examined. c-Jun is known to be upregulated by mechanical stress.^49^ c-Jun mRNA expression was proportionally upregulated by compression (Supplementary Figure S1H). The deformation of agarose gel and alginate bead can affect porosity, which reduces the diffusion of nutrients and oxygen. The change in the supply of nutrients and oxygen causes alterations in gene expression.^50, 51^ In our model, the diffusibility of Ponceu S (MW:~750 Da) in 2% agarose gel was not different at all RCUs (0.3 cm/h) (Supplementary Figure S1C). The molecular weight of nutrients in the media used for this study was below 500 Da. At 10 RCU, the height of agarose gel was 0.5-fold decreased compared with that of control (at 0 RCU) (Supplementary Figure S1A). Therefore, we assumed that the porosity of agarose gel at 10 RCU may be similar with that of 4% agarose gel without compression. The diffusibility of Ponceu S was not different in 4% agarose gel without compression (Supplementary Figure S1C). Alginate bead didn't show any difference in diffusibility at all RCUs (data not shown).

### Quantitative real-time PCR assays

Total and small RNAs were extracted with Trizol (Invitrogen; Carlsbad, CA, USA) and a miRNeasy Kit (Qiagen; Valencia, CA, USA), respectively. Total RNA was reverse transcribed using the TOPscript cDNA Synthesis Kit (Enzynomics; Daejeon-si, South Korea). MicroRNA was first modified by the addition of a poly (A) tail using *Escherichia coli* poly(A) polymerase and then reverse transcribed using a universal reverse transcription (RT) primer, as described previously.^52^ The resulting cDNA (25 ng) was amplified using the QuantiTect SYBR Green PCR Kit (Qiagen) for quantitative real-time PCR. PCR experiments were performed three times, each time in triplicate (totally nine). Primer sequences, melting temperature (Tm), and the product size are shown in Supplementary Table S1. Reverse transcription and real-time PCR were performed in a CFX Connect Real-Time PCR Detection System (Bio-Rad Laboratories; Hercules, CA, USA). The expression of gene transcripts was normalized to the geomean values of endogenous glyceraldehyde-3-phosphate dehydrogenase (*GAPDH*), succineate dehydrogenase complex subunit A, flavoprotein (FP)(SDHA), and hypoxanthine phosphoribosyltransferase 1 (HPRT1),^53^ and relative amounts were calculated according to the ΔΔCt method using CFX Manager Software (Bio-Rad).

### Bisulfite conversion and methylation-specific qPCR analysis

Genomic DNA was extracted from uncompressed, compressed CAFs, MDA-MB-231, and BT-474 using an Expin Gel SV Kit (GeneAll Biotechnology, Seoul, South Korea). For bisulfite conversion of unmethylated CpGs, 4 *μ*g of gDNA was modified with sodium bisulfite using an EpiTect Bisulfite Kit (Qiagen) according to the manufacturer's manual. For methylation-specific qPCR analysis, methylated DNA-specific and unmethylated DNA-specific primer sets were designed by analyzing CpG islands in the 2 kb upstream of mir-9-1, -2, and -3 using MethPrimer,^54^ as shown in Supplementary Figure S3 and Supplementary Table S2. Relative methylation was calculated using the ΔΔCt method. The Ct values of methylated DNA were normalized to those of unmethylated DNA.

### Firefly luciferase reporter constructs and luciferase assays

The wild-type 3'-UTRs of human *LAMC2*, *ITGA6*, *ITGB4*, and *EIF4E* were amplified by PCR using CAF genomic DNA as template. Mutant 3'-UTRs of each gene with deletions of the miR-9 seed sequence (from 7 to 10 bp) were generated by an overlap extension PCR method.^55^ Wild-type and mutant 3'UTRs were inserted downstream of the firefly luciferase-coding gene at the XbaI site of the pGL3 control vector (Supplementary Figure S6). The integrity and orientation of the inserts were confirmed by sequencing. For luciferase assays, transfection mixtures containing 200 ng of firefly luciferase reporter plasmid, 10 ng of pRL-TK (Renilla luciferase) (Promega; Madison, WI, USA) and 500 ng of miR-9 or control miR plasmid were transfected into 293T (2 × 10^5^) cells in six-well plates using Lipofectamine LTX with Plus Reagent (Invitrogen). The cells were harvested 48 h after transfection, and luciferase activity was measured using a dual-luciferase reporter assay system (Promega).

### Fluorescent imaging of miR-9 in compressed cells

In the presence of the human Cy5-conjugated miR-9-5p probe (EMD Millipore; Billerica, MA, USA), the cell-alginate beads were compressed with 1 RCU for 24 h, or were incubated for additional 24 h without compression for the decompression studies. For the control, the cell-alginate beads were cultured for 24 h without compression. The cells were isolated from the alginate beads by depolymerization, fixed with 4% paraformaldehyde, smeared onto a gelatin-coated microscope slide, and then mounted with a coverslip and mounting medium (Abcam). Fluorescent images were taken using a NIKON ECLIPSE Ti (Nikon Instruments, Inc.; Melville, NY, USA). Fluorescence images were quantified by measuring red-colored area using Image J (Bethesda, MD, USA) (version 1.50i).

### *In situ* proximity ligation assay (PLA)

After deparaffinization and rehydration, the slides with tissue sections (4 *μ*m) were washed twice with PBS and then blocked for 1 h at RT with 2% (w/v) BSA in PBS containing 0.1% (v/v) Triton X100. The slides were subsequently incubated at 4 °C overnight with anti-ITGA6 (1:100 dilution) (Abcam) and anti-ITGB4 (1:100) (Santa Cruz Biotechnology; Dallas, TX, USA) antibodies for double recognition or anti-VEGFA antibody (1:100) (Santa Cruz) for single recognition in a humidity chamber. Probe ligation was performed using DUOlink (OLINK Biosciences; Uppsala, Sweden), as recommended by the manufacturer. Images were taken using a LSM710 confocal laser scanning microscope and ZEN software (Carl Zeiss; Oberkochen, Germany). Fluorescence images were quantified by measuring red-colored area using Image J (version 1.50i).

### Treatment with anti-miR-9

Cells were cultured in six-well plates until 70% confluent, and then transfected with 0.1 *μ*M of anti-miR-9 or negative control miR for 24 h using G-Fectin transfection reagent (Gemolution Pharmaceuticals, Inc.; Seoul, South Korea).

### Western blot

For protein extraction, cells were lysed in 50 *μ*l of PRO-PREP Protein Extraction Solution (iNtRON Biotechnology; Seongnam-si, South Korea), homogenized using a 30-gauge needle, incubated for 30 min at 4 °C and then separated by centrifugation at 15 000 × *g*. After quantifying proteins in the extracts using the Bradford method, 20 *μ*g protein was subjected to electrophoresis on 10% polyacrylamide gels in Tris/glycine (Invitrogen), transferred to a PVDF membrane (Millipore) and then probed with primary antibodies against LAMC2, ITGA6, ITGB4, EIF4E, VEGFA, DNMT3B, DNMT3L, and GAPDH (Santa Cruz), and DNMT3A (Abcam). Primary antibodies were detected by horseradish peroxidase-conjugated secondary antibodies (Invitrogen) and visualized using enhanced chemiluminescence reagents (Santa Cruz). The intensity of western bands were quantified using Image J. Each band was normalized with GAPDH and then presented as the relative intensity value of control- *versus* compressed sample.

### Chromatin immunoprecipitation assay

Chromatin immunoprecipitation (Chip) assay was performed with EpiQuick Chromatin immunoprecipitation kit (Epigentek, Farmingdale, NY, USA) according to the manufacturer's manual. Briefly, CAF-alginate beads were compressed at the RCU of 1 for 24 h, depolymerized with 102 mm EDTA. After being washed twice with PBS using centrifugation (1000 r.p.m. for 5 min), the CAFs were resuspended with the fresh culture medium containing 1% formaldehyde (final concentration) and incubated at room temperature (RT) for 10 min on a rocking platform (50–100 rpm) for fixation. The fixed CAFs were washed three times with ice-cold PBS, lyzed with PRO-PREP Protein Extraction Solution, and then centrifuged to collect supernatant. The supernatant was sonicated for DNA shearing, transferred to the anti-DNMT3A antibody (Abcam)-coated well of 96-well plate, incubated at RT for 1 h. For DNA elution, after washing PBS, proteinas K was treated to each well and incubated at 65 °C for 90 min. DNA was collected using spin column, and analyzed by real-time PCR. Relative binding to DNMT3A was calculated using the ΔΔCt method. The Ct values of DNMT3A-bound DNA were normalized to those of input DNA.^56^

### Statistical analysis

Statistical significance was determined using a Student's *t*-test and ANOVA. Results were considered to be significant at *P*<0.05. All statistical analyses were performed using Prism 6 for Windows (GraphPad Software, Inc.; La Jolla, CA, USA).

## References

[bib1] Li YS, Haga JH, Chien S. Molecular basis of the effects of shear stress on vascular endothelial cells. J Biomech 2005; 38: 1949–1971. 16084198 10.1016/j.jbiomech.2004.09.030

[bib2] Resnick N, Yahav H, Shay-Salit A, Shushy M, Schubert S, Zilberman LC et al. Fluid shear stress and the vascular endothelium: for better and for worse. Prog Biophys Mol Biol 2003; 81: 177–199. 12732261 10.1016/s0079-6107(02)00052-4

[bib3] Traub O, Berk BC. Laminar shear stress - mechanisms by which endothelial cells transduce an atheroprotective force. Arterioscl Throm Vas 1998; 18: 677–685. 10.1161/01.atv.18.5.677 9598824

[bib4] Kazmierczak P, Sakaguchi H, Tokita J, Wilson-Kubalek EM, Milligan RA, Muller U et al. Cadherin 23 and protocadherin 15 interact to form tip-link filaments in sensory hair cells. Nature 2007; 449: 87–U59. 17805295 10.1038/nature06091

[bib5] Sumpio BE, Banes AJ, Levin LG, Johnson G. Mechanical-stress stimulates aortic endothelial-cells to proliferate. J Vasc Surg 1987; 6: 252–256. 3625881

[bib6] Yamaguchi M, Aihara N, Kojima T, Kasai K. RANKL increase in compressed periodontal ligament cells from root resorption. J Dent Res 2006; 85: 751–756. 16861294 10.1177/154405910608500812

[bib7] Farge E. Mechanical induction of twist in the *Drosophila* foregut/stomodeal primordium. Curr Biol 2003; 13: 1365–1377. 12932320 10.1016/s0960-9822(03)00576-1

[bib8] Ragan PM, Chin VI, Hung HHK, Masuda K, Thonar EJMA, Arner EC et al. Chondrocyte extracellular matrix synthesis and turnover are influenced by static compression in a new alginate disk culture system. Arch Biochem Biophys 2000; 383: 256–264. 11185561 10.1006/abbi.2000.2060

[bib9] Loening AM, James IE, Levenston ME, Badger AM, Frank EH, Kurz B et al. Injurious mechanical compression of bovine articular cartilage induces chondrocyte apoptosis. Arch Biochem Biophys 2000; 381: 205–212. 11032407 10.1006/abbi.2000.1988

[bib10] Hanahan D, Weinberg RA. Hallmarks of cancer: the next generation. Cell 2011; 144: 646–674. 21376230 10.1016/j.cell.2011.02.013

[bib11] Stylianopoulos T, Martin JD, Snuderl M, Mpekris F, Jain SR, Jain RK. Coevolution of solid stress and interstitial fluid pressure in tumors during progression: implications for vascular collapse. Cancer Res 2013; 73: 3833–3841. 23633490 10.1158/0008-5472.CAN-12-4521 PMC3702668

[bib12] Paszek MJ, Weaver VM. The tension mounts: mechanics meets morphogenesis and malignancy. J Mammary Gland Biol Neoplasia 2004; 9: 325–342. 15838603 10.1007/s10911-004-1404-x

[bib13] Stylianopoulos T, Martin JD, Chauhan VP, Jain SR, Diop-Frimpong B, Bardeesy N et al. Causes, consequences, and remedies for growth-induced solid stress in murine and human tumors. Proc Natl Acad Sci USA 2012; 109: 15101–15108. 22932871 10.1073/pnas.1213353109 PMC3458380

[bib14] Paszek MJ, Zahir N, Johnson KR, Lakins JN, Rozenberg GI, Gefen A et al. Tensional homeostasis and the malignant phenotype. Cancer Cell 2005; 8: 241–254. 16169468 10.1016/j.ccr.2005.08.010

[bib15] Levental KR, Yu H, Kass L, Lakins JN, Egeblad M, Erler JT et al. Matrix crosslinking forces tumor progression by enhancing integrin signaling. Cell 2009; 139: 891–906. 19931152 10.1016/j.cell.2009.10.027 PMC2788004

[bib16] Tse JM, Cheng G, Tyrrell JA, Wilcox-Adelman SA, Boucher Y, Jain RK et al. Mechanical compression drives cancer cells toward invasive phenotype. Proc Natl Acad Sci USA 2012; 109: 911–916. 22203958 10.1073/pnas.1118910109 PMC3271885

[bib17] Kim BG, An HJ, Kang S, Choi YP, Gao MQ, Park H et al. Laminin-332-rich tumor microenvironment for tumor invasion in the interface zone of breast cancer. Am J Pathol 2011; 178: 373–381. 21224074 10.1016/j.ajpath.2010.11.028 PMC3069863

[bib18] Marinkovich MP. Tumour microenvironment: laminin 332 in squamous-cell carcinoma. Nat Rev Cancer 2007; 7: 370–380. 17457303 10.1038/nrc2089

[bib19] Kim BG, Gao MQ, Choi YP, Kang S, Park HR, Kang KS et al. Invasive breast cancer induces laminin-332 upregulation and integrin beta 4 neoexpression in myofibroblasts to confer an anoikis-resistant phenotype during tissue remodeling. Breast Cancer Res 2012; 14: R88. 22673183 10.1186/bcr3203 PMC3446351

[bib20] Mercurio AM, Rabinovitz I. Towards a mechanistic understanding of tumor invasion—lessons from the alpha6beta 4 integrin. Semin Cancer Biol 2001; 11: 129–141. 11322832 10.1006/scbi.2000.0364

[bib21] Chung J, Bachelder RE, Lipscomb EA, Shaw LM, Mercurio AM. Integrin (alpha 6 beta 4) regulation of eIF-4E activity and VEGF translation: a survival mechanism for carcinoma cells. J Cell Biol 2002; 158: 165–174. 12105188 10.1083/jcb.200112015 PMC2173018

[bib22] Lehmann U, Hasemeier B, Christgen M, Muller M, Romermann D, Langer F et al. Epigenetic inactivation of microRNA gene hsa-mir-9-1 in human breast cancer. J Pathol 2008; 214: 17–24. 17948228 10.1002/path.2251

[bib23] Iorio MV, Ferracin M, Liu CG, Veronese A, Spizzo R, Sabbioni S et al. MicroRNA gene expression deregulation in human breast cancer. Cancer Res 2005; 65: 7065–7070. 16103053 10.1158/0008-5472.CAN-05-1783

[bib24] Chen LJ, Wei SY, Chiu JJ. Mechanical regulation of epigenetics in vascular biology and pathobiology. J Cell Mol Med 2013; 17: 437–448. 23551392 10.1111/jcmm.12031 PMC3822644

[bib25] Mouw JK, Yui Y, Damiano L, Bainer RO, Lakins JN, Acerbi I et al. Tissue mechanics modulate microRNA-dependent PTEN expression to regulate malignant progression. Nat Med 2014; 20: 360. 24633304 10.1038/nm.3497 PMC3981899

[bib26] Holliday DL, Speirs V. Choosing the right cell line for breast cancer research. Breast Cancer Res 2011; 13: 215. 21884641 10.1186/bcr2889 PMC3236329

[bib27] Mio K, Kirkham J, Bonass WA. Tips for extracting total RNA from chondrocytes cultured in agarose gel using a silica-based membrane kit. Anal Biochem 2006; 351: 314–316. 16460656 10.1016/j.ab.2005.12.031

[bib28] Vogelstein B, Gillespie D. Preparative and analytical purification of DNA from agarose. Proc Natl Acad Sci USA 1979; 76: 615–619. 284385 10.1073/pnas.76.2.615 PMC382999

[bib29] Mio K, Saito S, Tomatsu T, Toyama Y. Intermittent compressive strain may reduce aggrecanase expression in cartilage - a study of chondrocytes in agarose gel. Clin Orthop Relat R 2005; 433: 225–232. 10.1097/01.blo.0000150466.30696.c6 15805962

[bib30] Bougault C, Paumier A, Aubert-Foucher E, Mallein-Gerin F. Molecular analysis of chondrocytes cultured in agarose in response to dynamic compression. BMC Biotechnol 2008; 8: 71. 18793425 10.1186/1472-6750-8-71 PMC2556324

[bib31] Smidsrod O, Skjakbraek G. Alginate as immobilization matrix for cells. Trends Biotechnol 1990; 8: 71–78. 1366500 10.1016/0167-7799(90)90139-o

[bib32] Evans A, Armstrong S, Whelehan P, Thomson K, Rauchhaus P, Purdie C et al. Can shear-wave elastography predict response to neoadjuvant chemotherapy in women with invasive breast cancer? Br J Cancer 2013; 109: 2798–2802. 24169359 10.1038/bjc.2013.660 PMC3844913

[bib33] Murray-Zmijewski F, Slee EA, Lu X. A complex barcode underlies the heterogeneous response of p53 to stress. Nat Rev Mol Cell Biol 2008; 9: 702–712. 18719709 10.1038/nrm2451

[bib34] Jaalouk DE, Lammerding J. Mechanotransduction gone awry. Nat Rev Mol Cell Biol 2009; 10: 63–73. 19197333 10.1038/nrm2597 PMC2668954

[bib35] Aguirre JI, Plotkin LI, Gortazar AR, Millan MM, O'Brien CA, Manolagas SC et al. A novel ligand-independent function of the estrogen receptor is essential for osteocyte and osteoblast mechanotransduction. J Biol Chem 2007; 282: 25501–25508. 17609204 10.1074/jbc.M702231200

[bib36] Ma L, Young J, Prabhala H, Pan E, Mestdagh P, Muth D et al. miR-9, a MYC/MYCN-activated microRNA, regulates E-cadherin and cancer metastasis. Nat Cell Biol 2010; 12: 247–U252. 20173740 10.1038/ncb2024 PMC2845545

[bib37] Jones PA, Takai D. The role of DNA methylation in mammalian epigenetics. Science 2001; 293: 1068–1070. 11498573 10.1126/science.1063852

[bib38] Siegfried Z, Eden S, Mendelsohn M, Feng X, Tsuberi BZ, Cedar H. DNA methylation represses transcription *in vivo*. Nat Genet 1999; 22: 203–206. 10369268 10.1038/9727

[bib39] Mersman DP, Du HN, Fingerman IM, South PF, Briggs SD. Polyubiquitination of the demethylase Jhd2 controls histone methylation and gene expression. Genes Dev 2009; 23: 951–962. 19346402 10.1101/gad.1769209 PMC2675863

[bib40] Santos F, Hendrich B, Reik W, Dean W. Dynamic reprogramming of DNA methylation in the early mouse embryo. Dev Biol 2002; 241: 172–182. 11784103 10.1006/dbio.2001.0501

[bib41] Kim J, Kollhoff A, Bergmann A, Stubbs L. Methylation-sensitive binding of transcription factor YY1 to an insulator sequence within the paternally expressed imprinted gene, Peg3. Hum Mol Genet 2003; 12: 233–245. 12554678 10.1093/hmg/ddg028

[bib42] Kangaspeska S, Stride B, Metivier R, Polycarpou-Schwarz M, Ibberson D, Carmouche RP et al. Transient cyclical methylation of promoter DNA. Nature 2008; 452: 112–115. 18322535 10.1038/nature06640

[bib43] Humphrey JD, Dufresne ER, Schwartz MA. Mechanotransduction and extracellular matrix homeostasis. Nat Rev Mol Cell Biol 2014; 15: 802–812. 25355505 10.1038/nrm3896 PMC4513363

[bib44] Kharaishvili G, Simkova D, Bouchalova K, Gachechiladze M, Narsia N, Bouchal J. The role of cancer-associated fibroblasts, solid stress and other microenvironmental factors in tumor progression and therapy resistance. Cancer Cell Int 2014; 14: 41. 24883045 10.1186/1475-2867-14-41 PMC4038849

[bib45] Gao MQ, Kim BG, Kang S, Choi YP, Park H, Kang KS et al. Stromal fibroblasts from the interface zone of human breast carcinomas induce an epithelial-mesenchymal transition-like state in breast cancer cells *in vitro*. J Cell Sci 2010; 123: 3507–3514. 20841377 10.1242/jcs.072900

[bib46] Kim BG, Kang S, Han HH, Lee JH, Kim JE, Lee SH et al. Transcriptome-wide analysis of compression-induced microRNA expression alteration in breast cancer for mining therapeutic targets. Oncotarget 2016; 7: 27468–27478. 27027350 10.18632/oncotarget.8322 PMC5053664

[bib47] De Ceuninck F, Lesur C, Pastoureau P, Caliez A, Sabatini M. Culture of chondrocytes in alginate beads. Methods Molr Med 2004; 100: 15–22. 10.1385/1-59259-810-2:015 15280584

[bib48] Lee DA, Knight MM, Bolton JF, Idowu BD, Kayser MV, Bader DL. Chondrocyte deformation within compressed agarose constructs at the cellular and sub-cellular levels. J Biomech 2000; 33: 81–95. 10609521 10.1016/s0021-9290(99)00160-8

[bib49] Fitzgerald JB, Jin M, Dean D, Wood DJ, Zheng MH, Grodzinsky AJ. Mechanical compression of cartilage explants induces multiple time-dependent gene expression patterns and involves intracellular calcium and cyclic AMP. J Biol Chem 2004; 279: 19502–19511. 14960571 10.1074/jbc.M400437200

[bib50] Cousins RJ. Nutritional regulation of gene expression. Am J Med 1999; 106(1A): 20S–23S discussion 50S-51S. 10089110 10.1016/s0002-9343(98)00342-8

[bib51] Dang TS, Walker M, Ford D, Valentine RA. Nutrigenomics: the role of nutrients in gene expression. Periodontology 2000 2014; 64: 154–160. 24320962 10.1111/prd.12001

[bib52] Hurteau GJ, Spivack SD, Brock GJ. Potential mRNA degradation targets of hsa-miR-200c, identified using informatics and qRT-PCR. Cell Cycle 2006; 5: 1951–1956. 16929162 10.4161/cc.5.17.3133

[bib53] Vandesompele J, De Preter K, Pattyn F, Poppe B, Van Roy N, De Paepe A et al. Accurate normalization of real-time quantitative RT-PCR data by geometric averaging of multiple internal control genes. Genome Biol 2002; 3: RESEARCH0034. 12184808 10.1186/gb-2002-3-7-research0034 PMC126239

[bib54] Li LC, Dahiya R. MethPrimer: designing primers for methylation PCRs. Bioinformatics 2002; 18: 1427–1431. 12424112 10.1093/bioinformatics/18.11.1427

[bib55] Vallejo AN, Pogulis RJ, Pease LR. PCR mutagenesis by overlap extension and gene SOE. CSH Protoc 2008; 2008 pdb prot4861. 10.1101/pdb.prot4861 21356760

[bib56] Mukhopadhyay A, Deplancke B, Walhout AJ, Tissenbaum HA. Chromatin immunoprecipitation (ChIP) coupled to detection by quantitative real-time PCR to study transcription factor binding to DNA in *Caenorhabditis elegans*. Nat Protoc 2008; 3: 698–709. 18388953 10.1038/nprot.2008.38 PMC2681100

