# Supplementary material for: Mechanical compression induces VEGFA overexpression in breast cancer via DNMT3A-dependent miR-9 downregulation
Source: Cell Death Dis. 2017 Mar 2;8(3):e2646–. doi: 10.1038/cddis.2017.73 (PMC5386566; doi:10.1038/cddis.2017.73)
Supplement: Supplementary Tables [file cddis201773x1.doc]

**CDDIS-16-1096R**

**Supplementary Table S1. Primers for Real-time PCR analysis.**

| **Primer** | **Direction** | **Sequence (5’ to 3’)** | **Tm (℃)** | **Size (bp)** | **Product size (bp)** |
| --- | --- | --- | --- | --- | --- |
| LAMC2 | Forward | GATGGCATTCACTGCGAGAAG | 56.9 | 21 | 105 |
|  | Reverse | TCGAGCACTAAGAGAACCTTTGG | 56.5 | 23 |  |
| ITGA6 | Forward | TCATGGATCTGCAAATGGAA | 60.0 | 20 | 135 |
|  | Reverse | AGGGAACCAACAGCAACATC | 59.9 | 20 |  |
| ITGB4 | Forward | GTCTTCTCCACCGAGTCAGC | 53.3 | 20 | 138 |
|  | Reverse | CGGGTAGTCCTGTGTCCTGT | 53.5 | 20 |  |
| EIF4E | Forward | GGATGGTATTGAGCCTATGT | 54.7 | 20 | 104 |
|  | Reverse | AAAAGCGATCGAGGTCACTT | 52.8 | 20 |  |
| VEGFA | Forward | TTGTACAAGATCCGCAGACG | 59.8 | 20 | 100 |
|  | Reverse | TCACATCTGCAAGTACGTTCG | 59.9 | 21 |  |
| DNMT3a | Forward | GGCATTCAGGTGGACCGCTA | 59.3 | 20 | 205 |
|  | Reverse | CCTTGCGAGCAGGGTTGACG | 62.3 | 20 |  |
| DNMT3b | Forward | GCAAAGACCGAGGGGATGAA | 58.4 | 20 | 100 |
|  | Reverse | CTGCCACAAGACAAACAGCC | 55.3 | 20 |  |
| DNMT3L | Forward | TGAGCAACTGGGTGTGCTAC | 53.2 | 20 | 107 |
|  | Reverse | CGACTCTCGGTCGTAGAAGG | 53.7 | 20 |  |
| GAPDH | Forward | TGCACCACCAACTGCTTAGC | 55.9 | 20 | 87 |
|  | Reverse | GGCATGGACTGTGGTCATGAG | 57.3 | 21 |  |
| SDHA | Forward | TGGGAACAAGAGGGCATCTG | 57 | 20 | 86 |
|  | Reverse | CCACCACTGCATCAAATTCATG | 57.3 | 22 |  |
| HPRT1 | Forward | TGACACTGGCAAAACAATGCA | 57.1 | 21 | 94 |
|  | Reverse | GGTCCTTTTCACCAGCAAGCT | 57 | 21 |  |
| miR-9 | Forward | TCTTTGGTTATCTAGCTGTATGA | 48 | 23 |  |
| mir-9-1 | Forward | CGGGGTTGGTTGTTATCTTTGGTTA | 60.8 | 25 | 89 |
|  | Reverse | TGGGGTTATTTTTACTTTCGGTTAT | 55.4 | 25 |  |
| mir-9-2 | Forward | GGAAGCGAGTTGTTATCTTTGGTTAT | 57.2 | 26 | 87 |
|  | Reverse | TGAAGGAGTTTTTACTTTCGGTTAT | 53.8 | 25 |  |
| mir-9-3 | Forward | GGAGGCCCGTTTCTCTCTTT | 56.0 | 20 | 90 |
|  | Reverse | TGAGAATCATTTCTACTTTCGGTTA | 53.2 | 25 |  |
| Universal PCR | Reverse | AACGAGACGACGACAGACTTT | 52.7 | 21 |  |
| URT |  | AACGAGACGACGACAGACTTTTTTTTTTTTTTT | 65.3 | 33 |  |

**Supplementary Table S2. Primers for methylation-specific PCR.**

| **Group** | **Name** | **Direction** | **Sequence (5’ to 3’)** | **Tm (℃)** | **Size (bp)** | **Product size (bp)** |
| --- | --- | --- | --- | --- | --- | --- |
| Methylated-specific | mir-9-1 | Forward | TTTTAGAGAAGGGTAGTGGAGATTC | 58.5 | 25 | 113 |
|  |  | Reverse | GCTAATCCCAAATAAAAAAAACG | 58.3 | 23 |  |
|  | mir-9-2 | Forward | TATTAGTACGGAGGAGGTAAAAGGTC | 59.5 | 26 | 129 |
|  |  | Reverse | GACAACGACAACAACAATAACGA | 60.0 | 23 |  |
|  | mir-9-3 | Forward | TTGGTCGATTTTTGGATTGAC | 59.8 | 21 | 112 |
|  |  | Reverse | CGCTTAAAAAACCTCGAACG | 59.9 | 20 |  |
| Unmethylated-specific | mir-9-1 | Forward | TTTAGAGAAGGGTAGTGGAGATTTG | 59.3 | 25 | 114 |
|  |  | Reverse | CCACTAATCCCAAATAAAAAAAACA | 59.6 | 25 |  |
|  | mir-9-2 | Forward | TTATATTAGTATGGAGGAGGTAAAAGGTT | 58.6 | 29 | 133 |
|  |  | Reverse | CAACAACAACAACAACAATAACAAC | 58.5 | 25 |  |
|  | mir-9-3 | Forward | ATTGGTTGATTTTTGGATTGATG | 60.0 | 23 | 116 |
|  |  | Reverse | AAACACTTAAAAAACCTCAAACACC | 59.0 | 25 |  |
