# Supplementary material for: Mechanical compression induces VEGFA overexpression in breast cancer via DNMT3A-dependent miR-9 downregulation
Source: Cell Death Dis. 2017 Mar 2;8(3):e2646–. doi: 10.1038/cddis.2017.73 (PMC5386566; doi:10.1038/cddis.2017.73)
Supplement: Supplementary Information [file cddis201773x2.docx]

**CDDIS-16-1096R**

**Supplementary Figure S1. Experimental model validation of agarose-scaffolded alginate bead culture for 3D static compression.**

Compression-dependent deformation of A) agarose gel and B) alginate beads. C) Dye diffusibility in the agarose gels compressed at different RCUs. 2% agarose molds with a well were made in 10-cm culture dish, compressed at different RCUs, and loaded with Ponceu S (MW: 750 Da) in the well. Diffusion of dye was measured at the three points every 1 hour up to 5 hours in three separated experiments. Dye diffusion in 4% agarose mold without compression was examined as a similar porous condition of 2% agarose molds at the RCU of 10. D) Cell viability at different compression conditions. The CAFs exposed to different compression loads (0, 0.5, 1, 2, 5, and 10 RCU) for 1 day were stained with a fluorescein isothicyanate (FITC)-conjugated anti-annexin V antibody. For the positive control of apoptotic cells, the CAFs cultured in normal growth medium were incubated in 30% ethanol for 20 minutes. E) H&E staining of agarose-scaffolded CAF-alginate beads. Agarose-scaffolded CAF-alginate beads were pre-cultured for 1 day, and then fixed with 4% paraformaldehyde. Pericellular matrix deposition is evident as a brown circle around CAFs (black arrowed). F) Peak pattern (electropherogram) and G) Migration pattern (electrophoretic trace) of the RNAs extracted from the CAF-alginate beads compressed at different RCUs for 24h. RNA quality was measured using Agilent’s 2000 Bioanalyzer System. H) Compression-dependent upregulation of c-Jun.

**Supplementary Figure S2. Compression-induced miR-9 upregulation in MCF7 and SK-BR-3.**

A) Compression-induced miR-9 upregulation of MCF7 and SK-BR-3. B) Fluorescent images of compression-induced miR-9 upregulation in MCF7 and SK-BR-3. The scale bar is 200 μm. For fluorescent imaging, cells were compressed at the RCU of 1 for 24 h in the presence of a Cy5-conjugated miR-9-5p probe. Fluorescence images were quantified by measuring red-colored area using Image J. Compression-induced precursor miR-9 upregulation in C) MCF7 and D) SK-BR-3. For the expression analysis of miR-9 and its precursors, cells were compressed at the indicated RCUs for 24h. Data are represented as mean ± SD. Statistical significance was determined using a control- versus compressed-sample t-test. *, **, and *** represent p values of 0.01 to 0.05, 0.001 to 0.01, and < 0.001, respectively.

**Supplementary Figure S3. CpG island analysis and primer binding sites for measuring promoter methylation.**

2kb upstream of the three precursor miR-9s was analyzed and the primer sets for methylated and unmethylated DNA were designed by MethPrimer.

**Supplementary Figure S4. Compression-induced expression alteration of DNMT3B and DNMT3L in CAF, MDA-MB-231, and BT-474.**

Compression-induced expression alteration of A) *DNMT3B* mRNA and B) *DNMT3L* mRNA in CAF, MDA-MB-231, and BT-474. Data are represented as the mean ± SD. Statistical significance was determined using a control- versus compressed-sample t-test. * and *** represent p values of 0.01 to 0.05 and < 0.001, respectively. C) Compression-induced expression alteration of DNMT3B and DNMT3L protein in CAF, MDA-MB-231, and BT-474. The cells were compressed with the indicated RCUs for 24h. CAF1 was used as a representative for CAF.

**Supplementary Figure S5. Compression-induced expression alteration of DNMT3A, DNMT3B, and DNMT3L in MCF7 and SK-BR-3.**

Compression-induced expression alteration of A) *DNMT3A* mRNA, B) *DNMT3B* mRNA, and C) *DNMT3L* mRNA in MCF7 and SK-BR-3. Data are represented as the mean ± SD. Statistical significance was determined using a control- versus compressed-sample t-test. * and *** represent p values of 0.01 to 0.05 and < 0.001, respectively. D) Compression-induced expression alteration of DNMT3A, DNMT3B, and DNMT3L protein in MCF7 and SK-BR-3. The cells were compressed with the indicated RCUs for 24h. The intensity of Western bands were quantified using Image J. Each band was normalized with GAPDH, and then presented as the relative intensity value of control -versus compressed sample.

**Supplementary Figure S6. Plasmid constructs of the 3’UTRs of VEGF production signaling-related miR-9 target genes.**

Wild and mutant types of the 3’UTRs of *LAMC2*, *ITGA6*, *ITGB4*, and *EIF4E* were cloned into pGL3 control vector. Mutant types were constructed by deleting the miR-9 seed sequence using overlap PCR.
