# Supplementary figures and images for: Mechanical compression induces VEGFA overexpression in breast cancer via DNMT3A-dependent miR-9 downregulation
Source: Cell Death Dis. 2017 Mar 2;8(3):e2646–. doi: 10.1038/cddis.2017.73 (PMC5386566; doi:10.1038/cddis.2017.73)

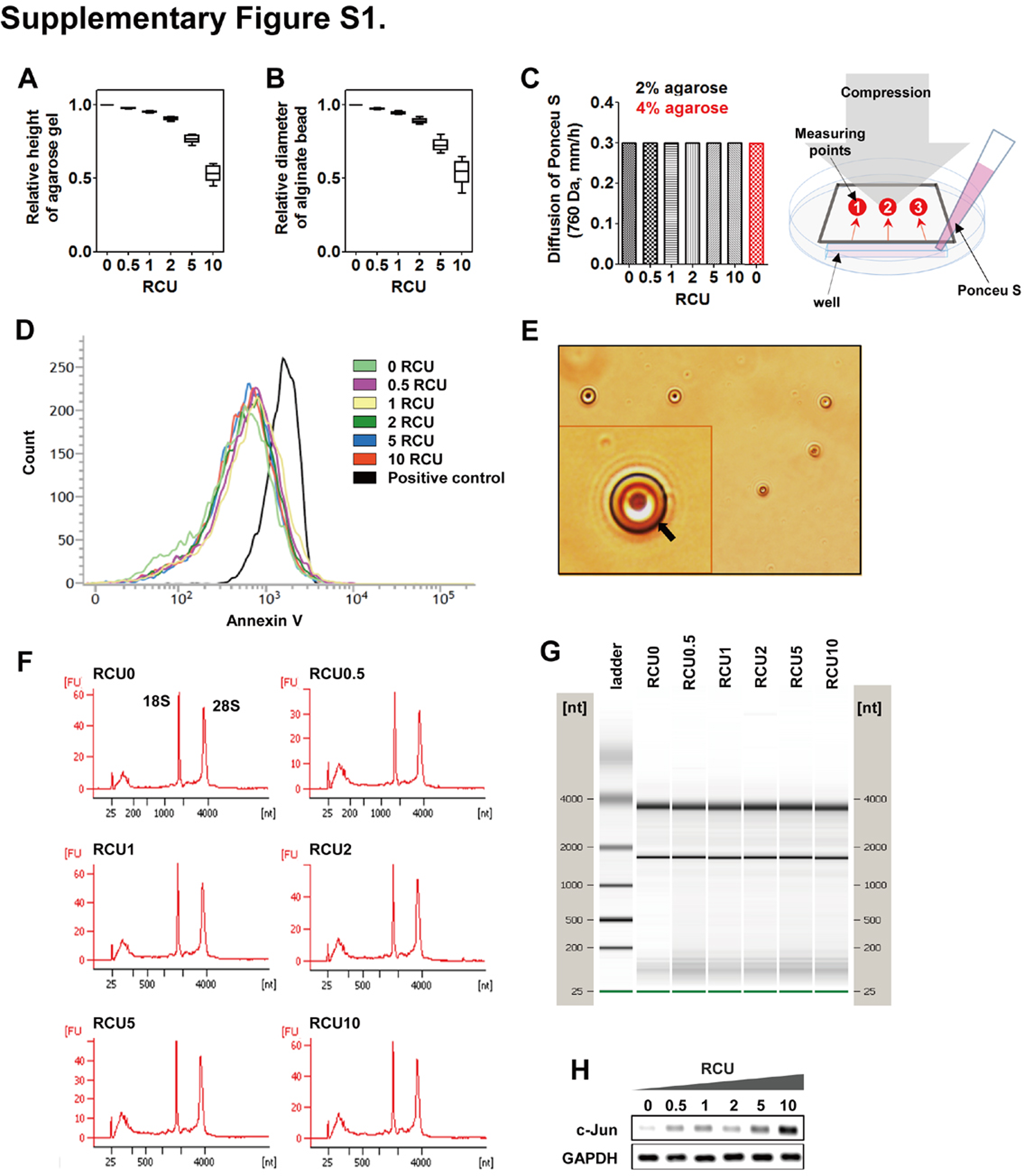

Supplement: Supplementary Figure S1 [file cddis201773x3.tif]

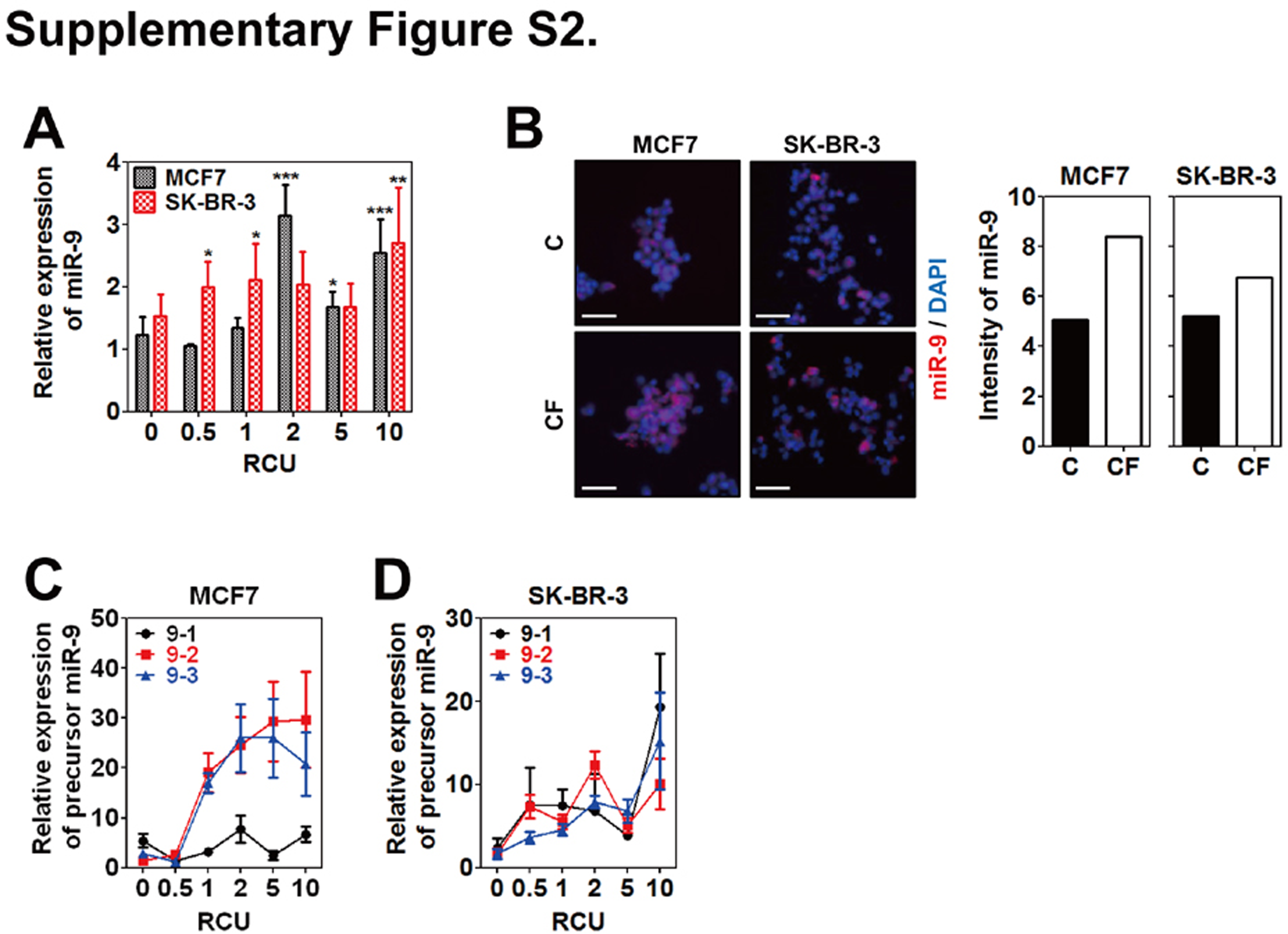

Supplement: Supplementary Figure S2 [file cddis201773x4.tif]

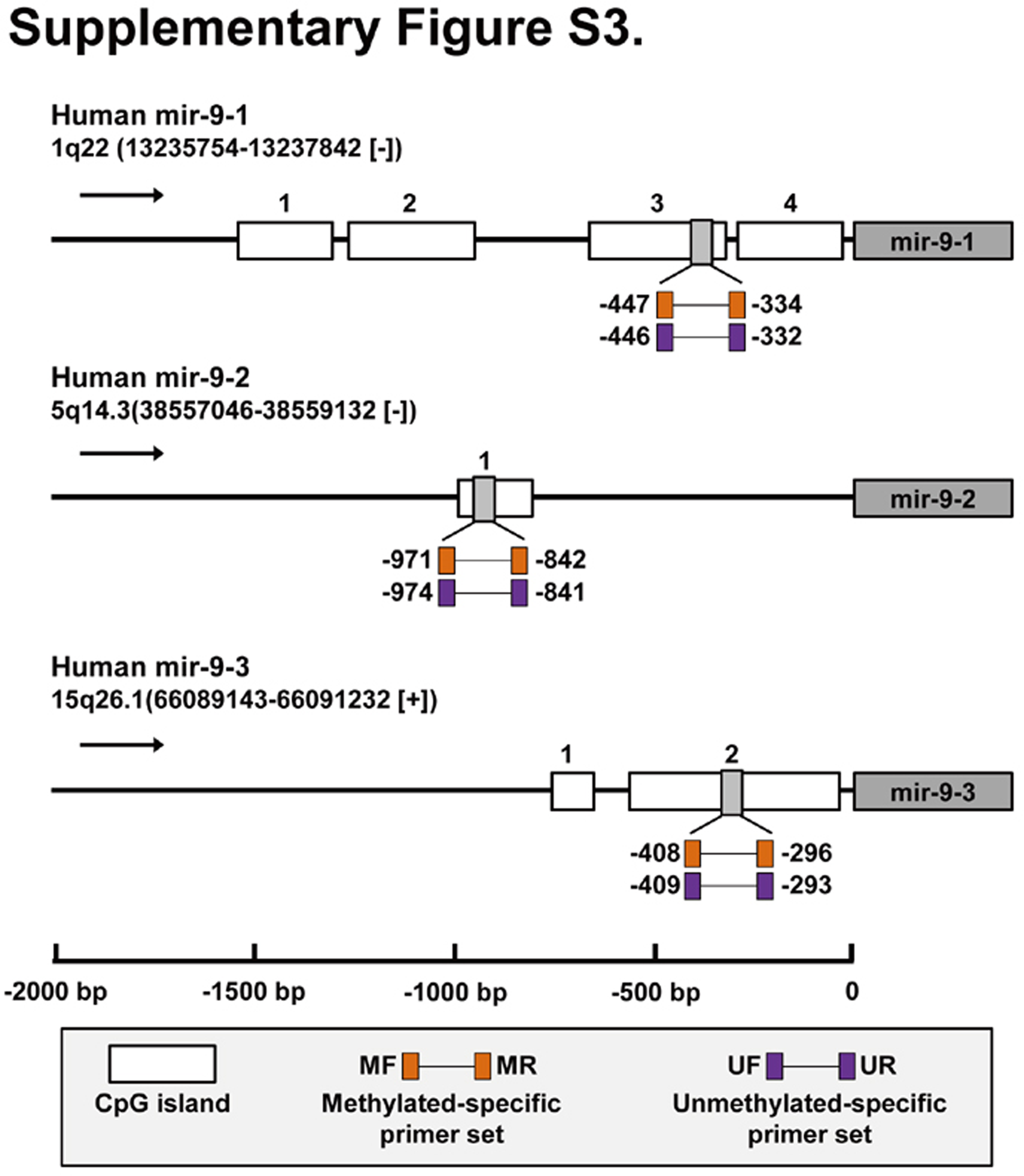

Supplement: Supplementary Figure S3 [file cddis201773x5.tif]

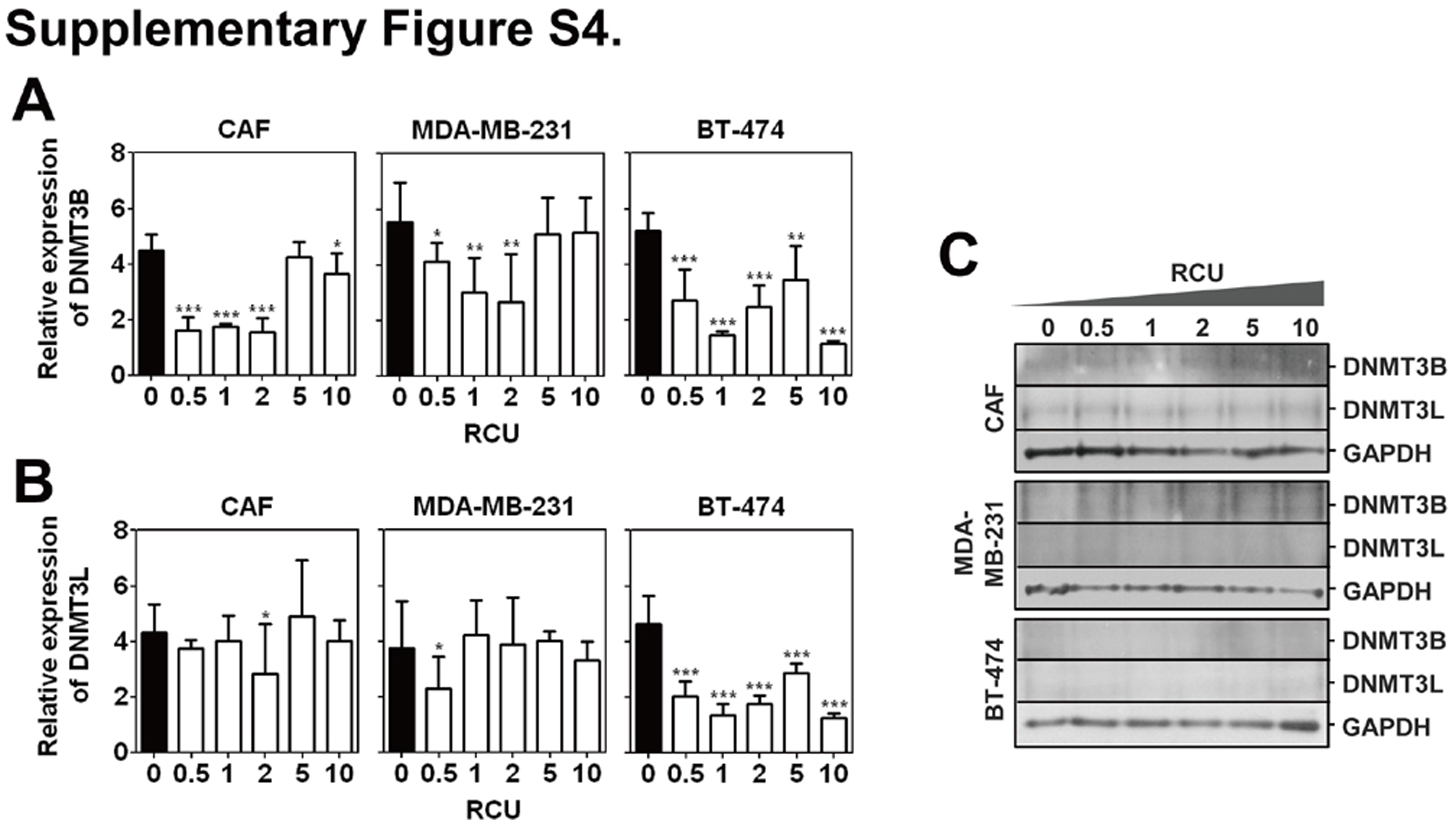

Supplement: Supplementary Figure S4 [file cddis201773x6.tif]

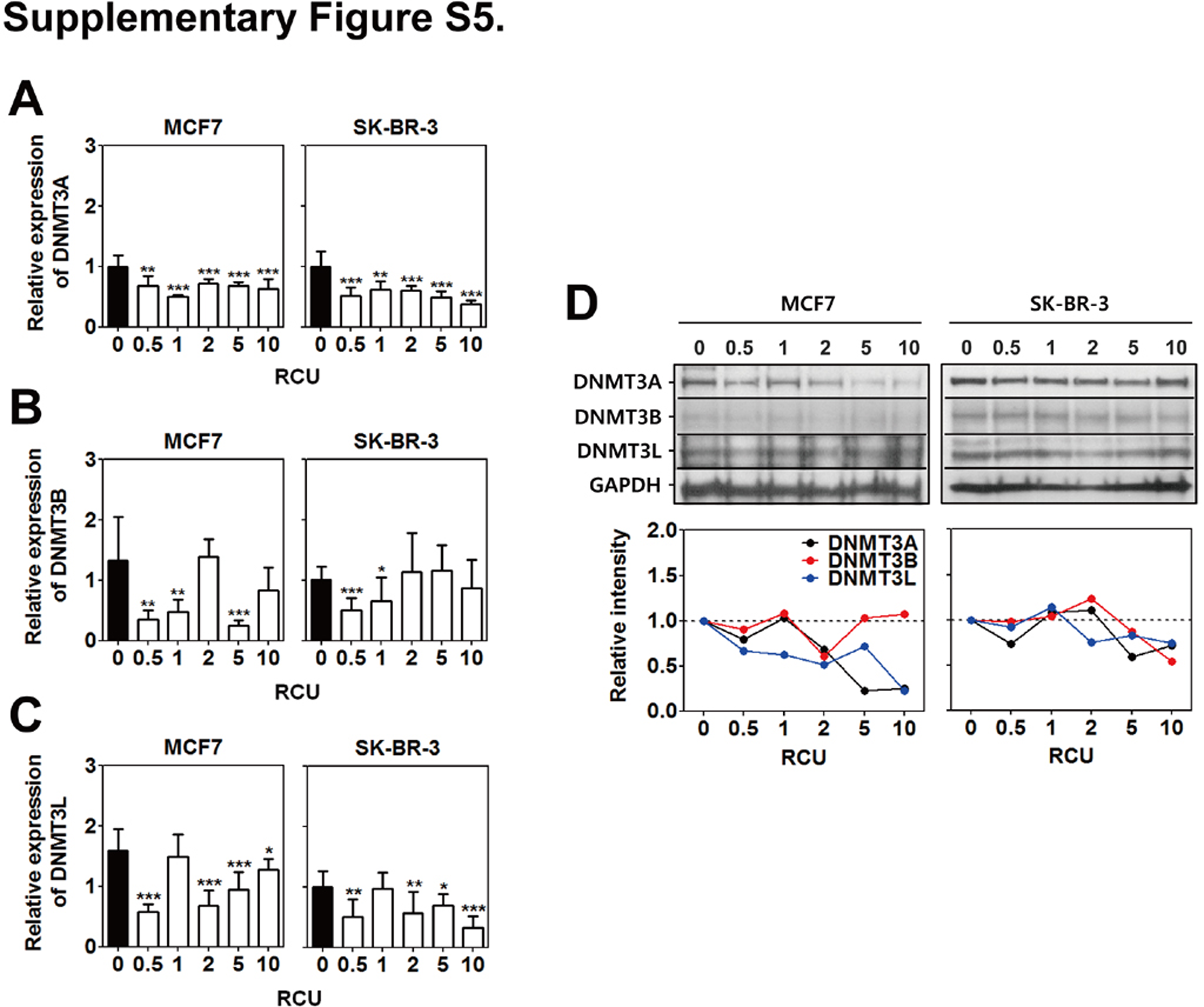

Supplement: Supplementary Figure S5 [file cddis201773x7.tif]

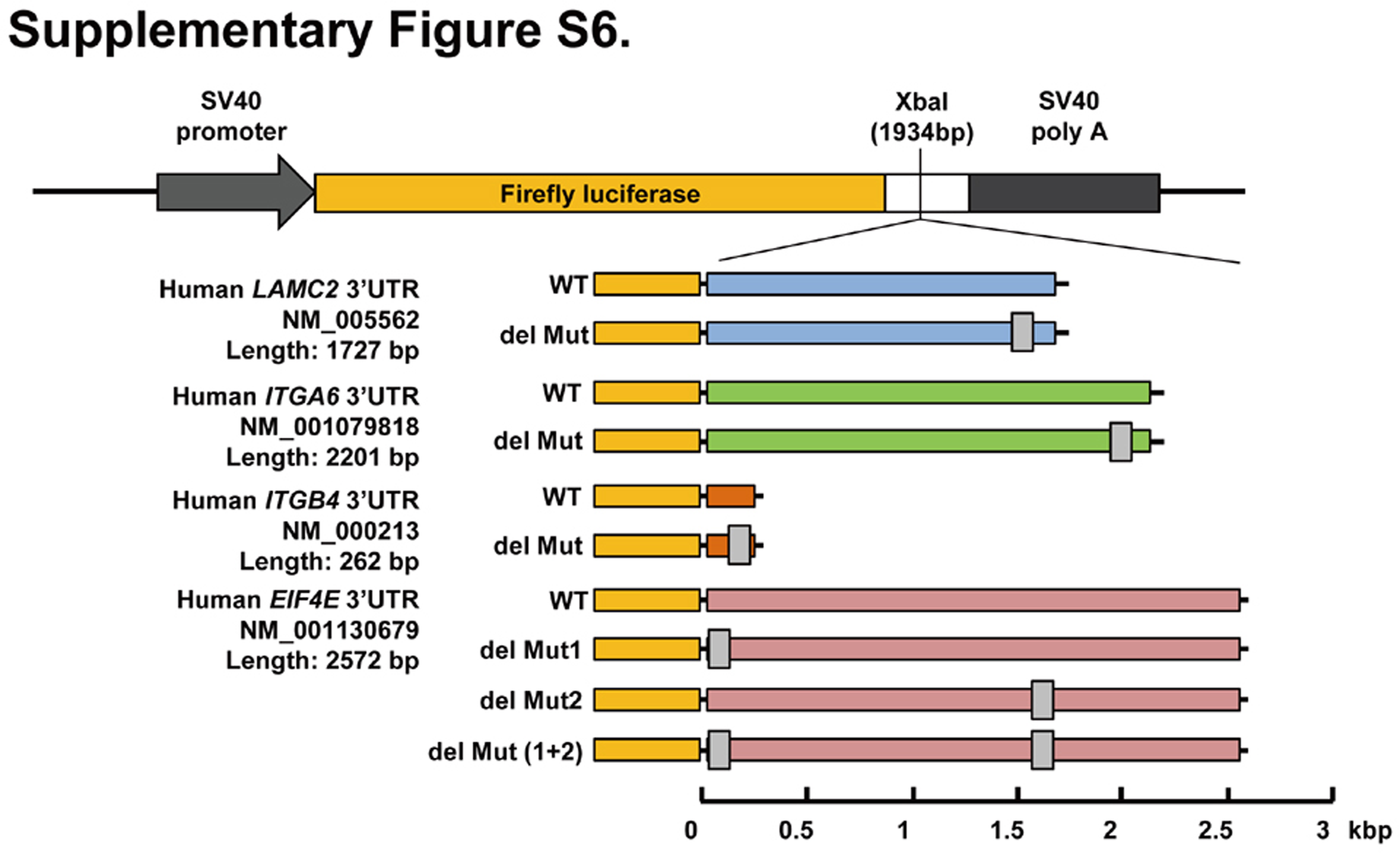

Supplement: Supplementary Figure S6 [file cddis201773x8.tif]
